# Using network analysis to model the effects of the SARS Cov2 pandemic on acute patient care within a healthcare system

**DOI:** 10.1038/s41598-022-14261-3

**Published:** 2022-06-16

**Authors:** Katharina Kohler, Matthew D. Jankowski, Tom Bashford, Deepi G. Goyal, Elizabeth B. Habermann, Laura E. Walker

**Affiliations:** 1 Department of Medicine, University of Cambridge, Cambridge, UK; 2 Department of Information Technology, Mayo Clinic, Rochester, MN USA; 3 Department of Medicine and Engineering, University of Cambridge, Cambridge, UK; 4 Department of Emergency Medicine, Mayo Clinic, Rochester, MN USA; 5 Kern Center for the Science of Healthcare Delivery, Mayo Clinic, Rochester, MN USA

**Keywords:** Health care, Health services

## Abstract

Consolidation of healthcare in the US has resulted in integrated organizations, encompassing large geographic areas, with varying services and complex patient flows. Profound changes in patient volumes and behavior have occurred during the SARS Cov2 pandemic, but understanding these across organizations is challenging. Network analysis provides a novel approach to address this. We retrospectively evaluated hospital-based encounters with an index emergency department visit in a healthcare system comprising 18 hospitals, using patient transfer as a marker of unmet clinical need. We developed quantitative models of transfers using network analysis incorporating the level of care provided (ward, progressive care, intensive care) during pre-pandemic (May 25, 2018 to March 16, 2020) and mid-pandemic (March 17, 2020 to March 8, 2021) time periods. 829,455 encounters were evaluated. The system functioned as a non-small-world, non-scale-free, dissociative network. Our models reflected transfer destination diversification and variations in volume between the two time points – results of intentional efforts during the pandemic. Known hub-spoke architecture correlated with quantitative analysis. Applying network analysis in an integrated US healthcare organization demonstrates changing patterns of care and the emergence of bottlenecks in response to the SARS Cov2 pandemic, consistent with clinical experience, providing a degree of face validity. The modelling of multiple influences can identify susceptibility to stress and opportunities to strengthen the system where patient movement is common and voluminous. The technique provides a mechanism to analyze the effects of intentional and contextual changes on system behavior.

## Introduction

The delivery of high-quality health care is an increasing challenge in the United States (US) as healthcare economics have led to consolidation of individual providers and smaller groups into larger systems^1^. Rural hospital and clinic closures have reduced local access and increased travel to larger centers^2^. While the impact of diversion from a single rural hospital may be easily absorbed by a large hub, patient movement from multiple smaller hospitals may ultimately overwhelm the capacity of receiving centers, prompting a need for additional beds^3^.

US healthcare enterprises include hospitals and clinics of varying sizes providing different services. Admissions via an Emergency Department (ED) present challenges to these organizations by creating a variety of potential care pathways dependent on patient and hospital characteristics. Additionally, these visits are by their nature unexpected and include a heterogenous population. Compared to elective admissions, emergency pathways of care are less-well delineated. This is further complicated by mismatches between the ED/hospital a patient chooses and the distribution of specialty services, potentially necessitating transfers between sites. Recently, the SARS Cov2 pandemic has impacted emergency admissions by altering the number of patients presenting, their clinical characteristics and the availability of healthcare services^4^. Understanding the patient flows that define utilization becomes crucial as health systems determine how best to strategically locate and scale resources to ensure patient access.

Healthcare organizations can be conceptualised as systems, which may exhibit complex behaviour, with the sum of these interactions resulting in system behaviour which is not easily predictable^5^. Complex systems may be studied using qualitative or quantitative techniques.

Network analysis provides an established quantitative methodological approach^6^ that has been used to describe a range of complex systems, from the world wide web^6^ to social networks^7^. In network science a complex system is represented as a set of components (nodes) that interact via connections (edges). This provides a method of visualization of the system of interest, allowing investigation of both its structure and function. The methodology has been used in a range of healthcare settings^8^, for example: to model disease outbreaks^9^, patient referral networks^10^, patient movement through acute care units^11,12^ and resource utilization by emergency surgery patients^13^.

Important concepts in network analysis are related both to individual nodes or to the overall system structure. Nodes can be described by their connectivity (degree), associations with other nodes (assortativity and clustering coefficients) and importance to the network (betweenness centrality). Overall structure can be described by parameters such as average shortest path, density (fraction of possible edges that exist), overall assortativity and clustering coefficients. The metrics are then combined to describe networks as small-scale, with high clustering and short path lengths that allow for efficient connections between nodes, or scale-free, where the degree distribution follows a power law and contains a significant number of highly connected hub nodes. Real-world networks are often conceived to be scale-free, and to exhibit complex behaviours such as non-linearity and path dependence, however this has been questioned^14^.

We aimed to understand changes to patient flow associated with the onset of the pandemic in 2020, in the context of a large US healthcare enterprise. We explored geographic areas where patients demonstrated long-distance travel to access care, which sites had many inter-hospital transfers, and where bottlenecks arose. As a stress test for the network science model, we describe the changes to patient flow in two distinct phases of the SARS CoV-19 pandemic and correlate this for face validity with regional systemic responses to the pandemic.

## Methods

### Setting

This study was set in a Midwest hospital system comprised of an academic hospital, three large community hub hospitals with specialty services, three mid-sized hospitals with inpatient and surgical services, and eleven critical access hospitals. The community hub hospitals are in three geographic regions referring to the academic center, located in a fourth region with a set of nearby hospitals that refer there preferentially as a regional hub. Critical access hospitals provide small acute inpatient units, have limited surgical capability, with on-site post-acute care and long-term acute care beds at some facilities. All sites offer emergency medical care, outpatient primary care and a variety of specialty clinics.

The organization serves over 1 million patients per year^15^, primarily the community population, with the academic center providing specialty care for local, regional, national and international patients. Transfers between sites are driven by need for specialty consultation/procedures, intensive care needs, or advanced imaging studies, and hospital capacity.

### Data

Data were extracted from a proprietary platform and spanned May 25, 2018, to March 8, 2021. Hospital-based encounters originating in an ED were eligible for inclusion. Records for patients who did not consent to using their medical records for research were excluded from the analysis. Patients with incomplete pathways encounters that represented a small proportion of overall visits, such as international patients, were excluded. Data cleaning was performed in Python^16^. Data use was approved via the Mayo Clinic Internal Review Board.

Patient locations were grouped using home zip codes, and encounter-specific journeys when a patient was transferred between hospitals during a single episode of care were created by merging visits. We linked encounters with a common patient identifier, less than 24 h apart and accompanied by an indication of a transfer in the electronic health record (EHR). Data were validated using timeline chronicling and cross-referenced with patient census reports derived from the EHR (Epic, Verona WI).

### Model and analysis

Our model used patient movement between locations as a representation of unmet resource need at the originating site. Patient locations were represented as nodes and their movement as edges. The NetworkX package in Python^17^ was utilized to transform our dataset into patient journeys, which were subsequently visualized with Cytoscape and further analyzed using RStudio^19^.

Hospital nodes were encoded by regional location and size: academic center (AC), regional hub (RH), stand-alone ED (EDSS), critical access hospital (CAH). To represent resource usage more granularly, we added the level of care: emergency department (ED), floor level care (WARD), intensive care (ICU), intermediate level care (PCU). This resulted in a composite name for each node, e.g. “RH1_ED” reflecting a regional hub ED. We anonymized labels for the starting and ending patient origin regions such as “M1” for a home and “MORT” for patients who passed away during their hospitalization.

Geographical distances were calculated using the GeoPy package in Python^20^ with edge attributes representing volume of traffic and distance, resulting in a weighted directed network. Edges with less than 0.0001% of the traffic in the network were removed to increase clarity and aid analysis.

We created three patient movement networks: an overall visualization of the system and two models that represent pre-pandemic (May25, 2018 to March 16, 2020) and mid-pandemic (March 17, 2020 to March 8, 2021) time points. We analyzed the network structure using degree distribution, associativity, density and clustering coefficients^21^. When assessing individual node characteristics, we used betweenness centrality to represent the importance of a node and degree/weighted degree to represent connectivity. The combination of these allowed us to identify both hubs and bottlenecks within the system^22,23^.

## Results

There were 829,455 unique ED visits within the enterprise that met inclusion criteria, with 68% of these encounters occurring prior to the start of the pandemic.

**Figure 1:**
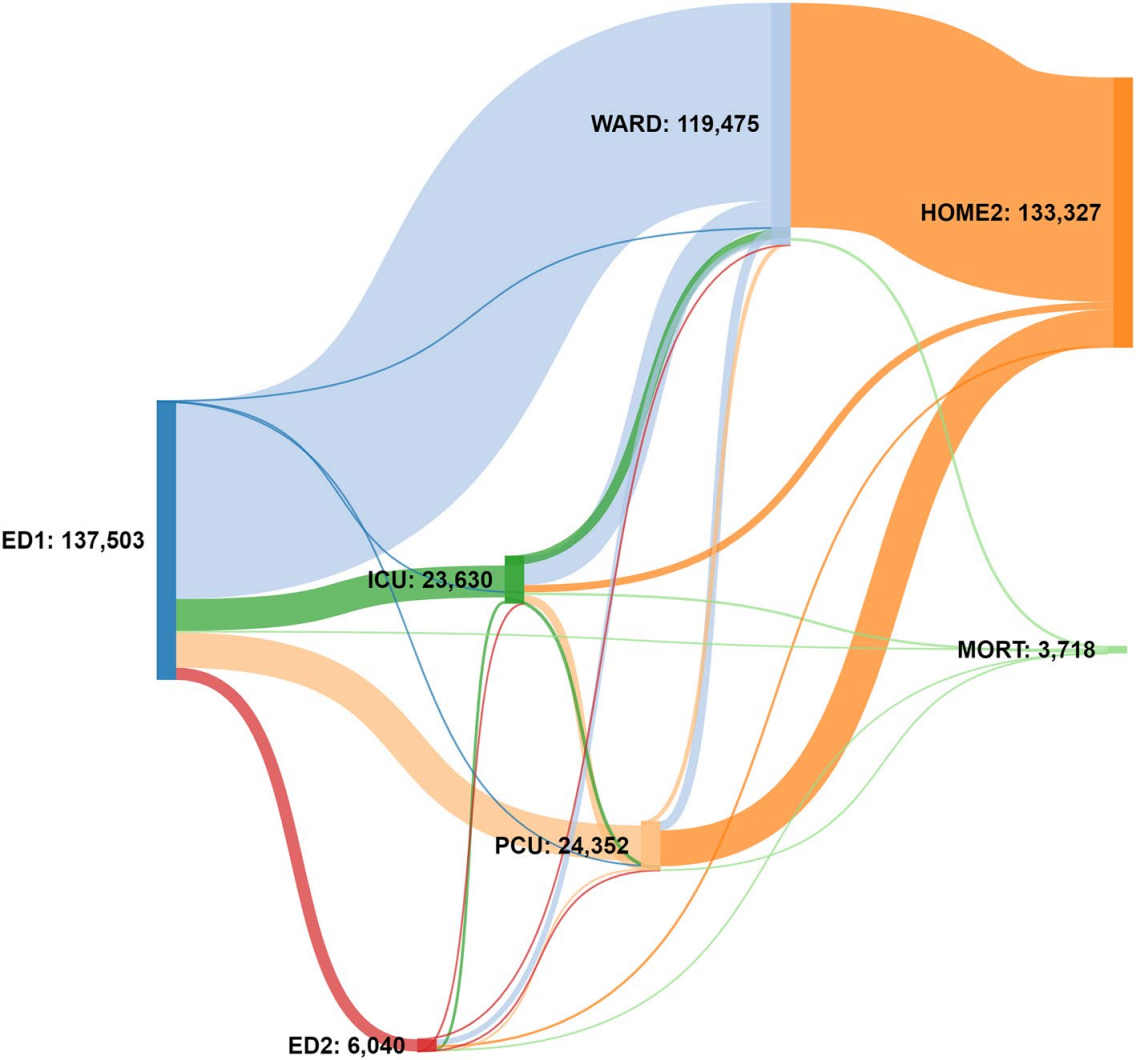
Sankey diagram visualizing the patient pathways. Pathways are colored by the receiving location and width of line represents the number of patients. All ED visits resulting in hospitalization, transfer or death are followed through the system to their endpoint. ED visits that resulted in discharge home directly from the initial ED attendance are excluded from this representation. Transfers to a further emergency department (ED2) are shown in red. The main journey of ED to ward to home is clearly displayed but with alternative ED to ICU or PCU to ward and then home contributing most of the remaining journeys.

A Sankey diagram (Fig. 1) was used to illustrate patient journeys, showing changes in the level of care for all patients in our study who were admitted, transferred, or died. The dominant patient journey was from ED to a ward admission followed by discharge home. An additional important pathway was ED to ICU/PCU, then ward admission, before discharge home. Inter-hospital transfers between EDs represented a small population in proportion to the whole system.

**Figure 2:**
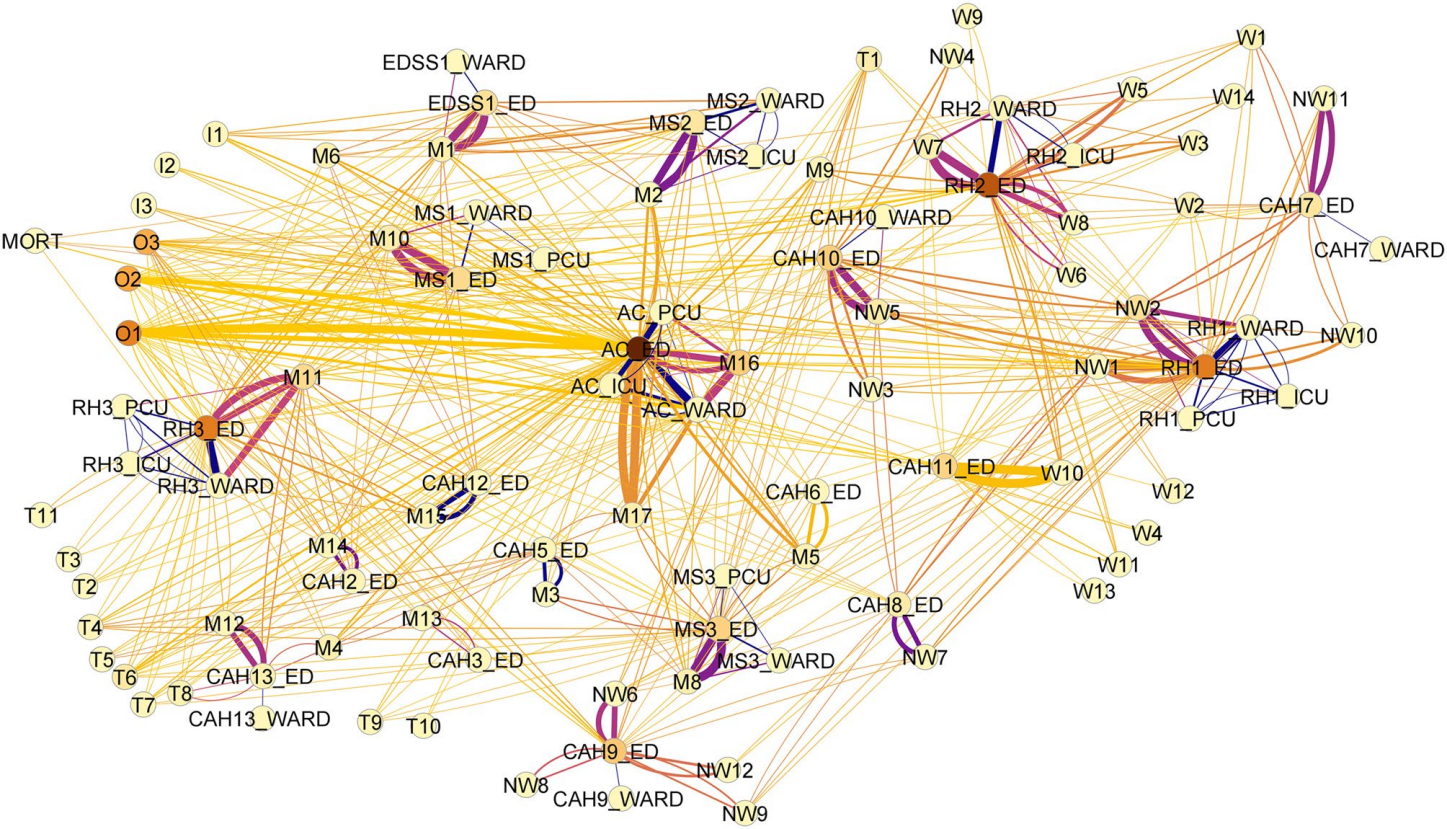
Patient movement network. Nodes represent hospital locations or patient home locations. Edge width indicates the traffic along an edge and edge color is related to the geographical distance between nodes with darker edges having shorter distance. Node color indicates the relative importance of the node to the network using betweenness centrality with darker nodes representing a higher betweenness centrality. The image highlights the extensive connectivity throughout the system with some local care preference evident.

The system-wide network analysis, including all patients with an emergency department presentation during the study period, is shown in Fig. 2**.** The data produced this graph included 1.598 million transfers with 101 nodes and 541 edges, after removal of very low-frequency transfers. The figure shows extensive interconnectedness between community locations and local hospitals, and with the academic center (AC), visible as a background of pale-yellow lines, that reflects patients seeking emergency care in diverse places. The importance and centrality of the AC is emphasized by the high number of connections received from a vast catchment area and its high betweenness centrality.

Unsurprisingly, the most-used pathways are the home nodes to the nearest hospital location. However, the model also reveals a complex system structure with significant connections across farther distances when patients bypass the local facility or transfer to a distant destination. As an example, the patient origin location M2 has a strong connection to its local hospital ED (MS2_ED), seen as a set of wide, dark purple lines between representing close proximity and a high volume. There is also a strong connection to AC_ED, represented by orange-yellow lines of moderate thickness. In this case, the M2 area represents the local community where the MS2 hospital is located. MS2 is a mid-sized hospital with limited specialty care and is situated near to AC, which serves as its specialty hub. Additional examples can be found with visual inspection.

Our model was found to have 101 nodes and 541 edges with a connection density of 0.054. We tested whether this system demonstrated the properties of a scale-free network, which would be resilient to random insults on nodes^24^**.** We found that the degree distribution had a heavy high degree tail, but that a log-normal degree distribution with fewer very high degree nodes than would be expected within a scale-free network. Small world parameters Ω^25^ and σ^26^ were 0.6 and 0.3 respectively, confirming the network to behave as a non-small world network. This places our system in contrast to scale-free small-world networks often grown organically by preferential attachment and that have specific robust characteristics^27^.

The average global clustering coefficient, reflecting the proportion of neighboring nodes that are also neighbors, was 0.19. This is much less than one would expect had the nodes attached randomly^28^. Therefore, locations that feed into a node are less likely than expected by random allocation to also feed into each other, reflecting the “hub and spoke” structure with patient origins (spokes) feeding into hospitals (hubs) and a fraction of connections transferring patients between spoke hospitals. This finding aligns with clinical observations, in which it is uncommon that a patient is transferred from a hospital that provides specialty care to one that does not, or for small hospitals to transfer patients between each other.

The network is a dissociative network (overall associativity coefficient of -0.5), where nodes with high degrees are connected to nodes with lower degrees. This confirms the hub and spoke structure with patient home locations connected to their local hospital ED. However, in contrast to a connected structure, this exposes the system to higher risk if one of the hubs has reduced capacity or experiences excess demand.

**Figure 3:**
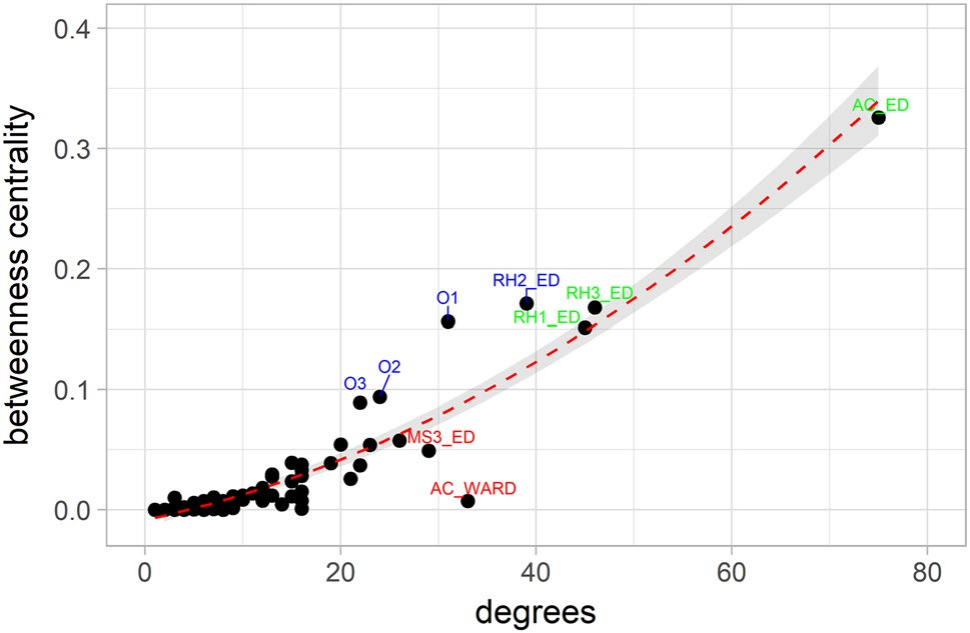
Relation of betweenness centrality and degree. The expected squared correlation is clearly visible with a few outliers. RH2_ED, O1, O2 and O3 (blue) show higher betweenness centrality than expected by their degree connectivity. These nodes, in particular RH2_ED (as a hospital location) are important for the integrity of the network. The other outliers, AC_WARD and MS3_ED (red), have betweenness centrality lower than expected. In green we show the major hubs of the network, AC_ED, RH1_ED and RH3_ED.

Numerical determination of hubs and bottlenecks using network parameters has been described in the literature^23^, where hubs are the nodes with the highest degree connectivity and bottlenecks are the nodes with the highest betweenness centrality. Additionally, betweenness centrality versus degree should follow a quadratic relationship^20^ (Fig. 3**)** with nodes above this curve considered bottlenecks. Both methods of determining hubs and bottlenecks agreed with few exceptions. Almost all hospital locations that are bottlenecks are also hubs (green): the EDs at all hubs (AC and RH1-3) have the highest degree and betweenness centrality. Two hospital nodes, AC_WARD and MS3_ED (red), are hubs but not bottlenecks; they are well-connected but less important to the system. Several of the home locations are pure bottlenecks, in particular, O1, O2, O3 nodes (blue) encompass a larger geographical area of patients that attend a variety of hospitals. RH2_ED was determined numerically to be both a hub and bottleneck, however the graphical analysis identified pure bottleneck characteristics.

The results of both hub/bottleneck determinations are in alignment with the clinical expectation that many patient pathways pass through an ED *en route* to AC_Ward and AC_ICU locations rather than non-ED locations. These nodes serve as hubs within the system without creating a significant bottleneck. O1 and O3, on the other hand, have limited connections due to proximity to their nearest facilities (RH3 and AC, respectively).

### Pre- and mid-pandemic analysis

Our chosen modelling timeframe encompasses pre-pandemic and mid-pandemic periods with the start of the pandemic set as March 18th 2020 to reflect changes in local conditions. This allows a description of the effect of changing health care provision, care-seeking behaviors, and disease incidences in our system during a critical time^4^.

Of the 829,455 patient encounters, ~ 1.1 million patient movements occurred pre-pandemic and 518,000 mid-pandemic. The two time points produced similarly structured networks. The pre-pandemic network having 111 nodes and a density of 0.18, and the mid-pandemic network having 107 nodes and a density of 0.15. Neither demonstrated small-world network characteristics, while both had a dissociative network structure (assortativity ~ − 0.4). With respect to workload in AC, ED connectivity did not change significantly (pre = 152 and post = 146), however connectivity for the ICU reduced from 35 to 29. Most other parameters for AC did not change significantly.

**Figure 4:**
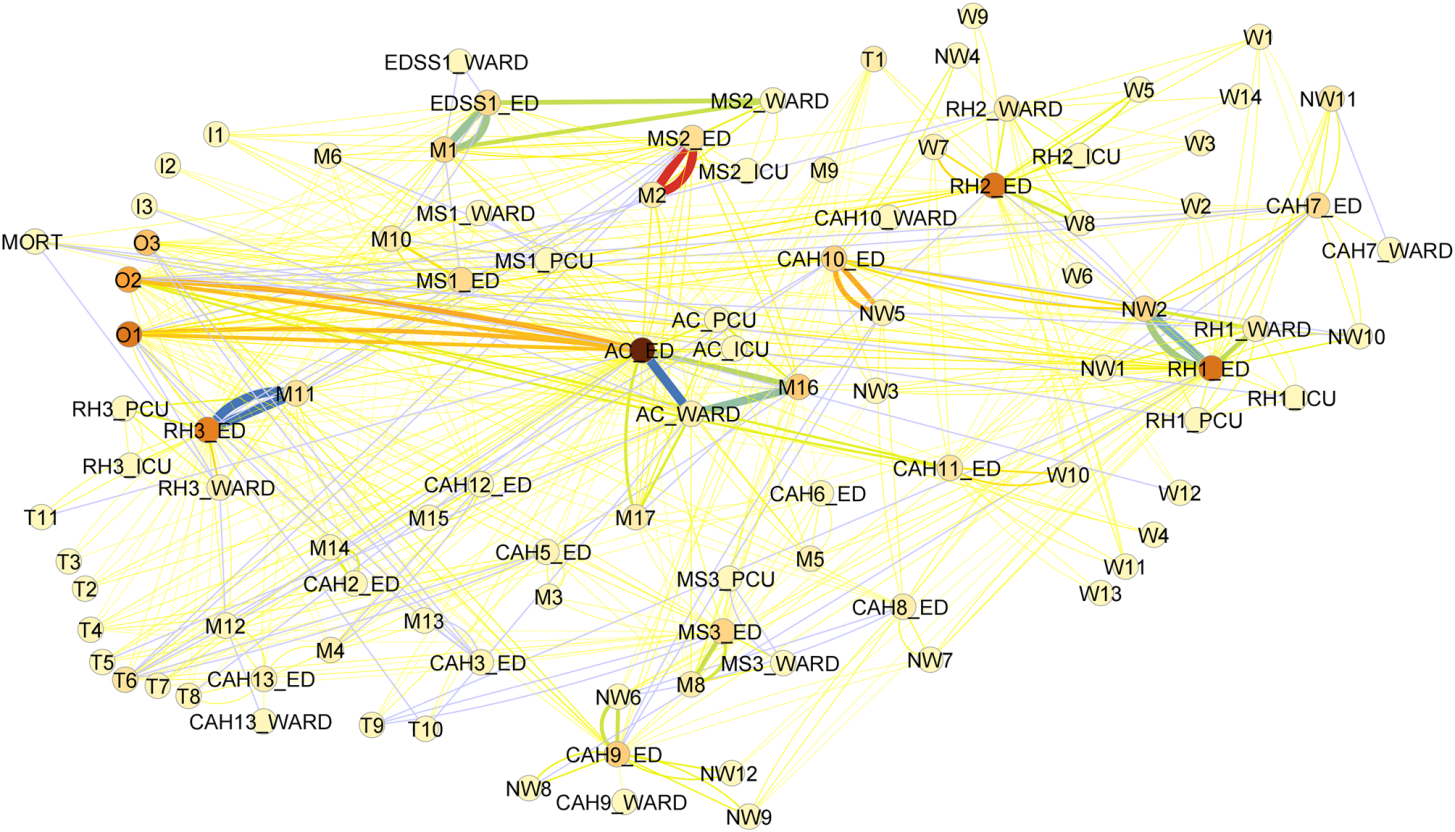
Difference network representing changes in patient movement between pre-and mid-pandemic. Node color represents degree connectivity (darker is higher) and increased edge weight/darker color represent a larger change between pre-and mid-pandemic.

We developed differential network models to represent changes in patient traffic between the two time periods (Fig. 4**)** with the same strategy for node coloration but edge weighting and color based on the magnitude of the difference between periods. Most connections exhibited slight changes in traffic, as shown by the extensive set of thin edges. Increased patient movement was seen within AC and from its local node M16. RH1 and RH3 saw increased patient presentations from NW2 and M11, respectively. There were increased transfers between EDSS1 and MS2, shown by the green edges connecting them (top of Fig. 3) and reduced patient attendance from M2, O1 and O2.

**Figure 5:**
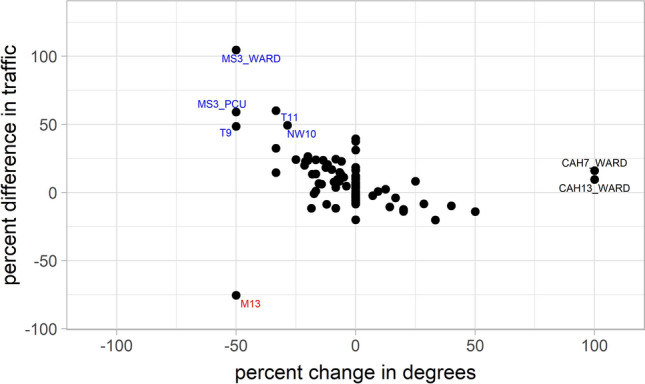
Change in node properties before and during the pandemic. The plot shows change in traffic through a node versus change in degree which represents connectivity to other hospitals or care locations. Two areas (CAH13_WARD and CAH7_WARD) significantly increased both their patient throughput and their connectivity whereas the M13 community reduced their patient supply to the system.

Changes in traffic and connectivity that occurred with the onset of the pandemic are visualized by showing percentage change in patient traffic through a node versus the percent change in degree connectivity (Fig. 5). Most nodes showed little change in degree connectivity and traffic, however there were reduced patient numbers from M13 while CAH13 and CAH7 wards had structural changes that increased their connectivity. Figure 5 showed the increase in traffic through the relatively low volume MS3 hospital (both ward and PCU) due to accounting for proportional changes and therefore highlighting more subtle changes in traffic.

## Discussion

We demonstrate successful representation of a regional US healthcare enterprise using network science methods based upon routinely collected electronic data. We used an established methodology applied to a novel setting, allowing exploration of system structure and function, detection of unexpected connections between system components, and analysis of patient flow changes following system perturbation caused by the SARS Cov2 pandemic.

Healthcare is a complex adaptive system which demonstrates behaviors such as non-linearity, non-scalability, and path dependence. This assertion has been supported through the application of network science, for example when applied to in-hospital patient flows^12^. However, in contrast to other systems, the overall architecture of the system in our study was shown to be that of a dissociative, non-small world, non-scale-free network. This finding, combined with the evident importance of the academic center as a key component and the traffic pattern through the three community hubs, likely reflects the intentional design of the system as opposed to one which evolved more organically over time. The patterns of patient movement within the system were similar to the authors’ expectations of patient care access patterns, providing a degree of face validity.

The interconnectivity of the whole network shows transportation of patients between sites is frequent. Interhospital transfers represent significant resource usage in terms of physical movement, and of clinical staff time. Complicated transportation logistics have important downstream effects, including the potential delay to definitive care for time-critical conditions^29–31^. Identifying patterns of connectivity can guide allocation of ambulance resources, changes to local service offerings, and other factors that determine where and how a patient can receive care.

The SARS Cov2 pandemic has posed a challenge to healthcare systems worldwide. Our analysis shows that the hub-and-spoke model was retained both pre- and mid-pandemic. This may be taken as a marker of ongoing system resilience. Patient flows did not show emergent complex behaviour creating unexpected hubs, bottlenecks or a new structure under the system stresses of the pandemic.

Mid-pandemic, efforts to reduce transfers between sites were enacted in anticipation of potential surge, in concert with an unexpected reduction in overall traffic through the EDs, is reflected in the decrease in patient movement from CAH and MS hospitals to RH and AC. Additionally, transfers between non-hub sites (MS1 and MS2, e.g.) were facilitated by a centralized transfer center, resulting in new connections.

The effects of changes to healthcare delivery are readily apparent in the network analysis, making this a tool to understand the current state and a way to monitor changes in a large system. Opportunities to improve patient-centered care can be identified using this analysis. Notably, patients travel long distances for care, sometimes bypassing a local ED in favor of presenting to a RH or AC. Qualitative exploration of this observed effect represents an opportunity to strengthen local care and better meet community needs. Rural hospitals are major local employers, and loss of economic stability due to poor utilization risks a devastating community event^32^.

## Conclusion

We demonstrate the application of network science, using routinely collected electronic data, to model the patient flow of a major US healthcare enterprise at both pre-pandemic and mid-pandemic time points. The resulting models reflect known system changes and are consistent with clinical experience. Network analysis provides insights into the system under study: it is planned rather than emergent; dissociative and at risk if key hubs fail; and contains identifiable bottlenecks.

In terms of reflecting changes due to the SARS Cov2 pandemic, our models show changes in patient flow consistent with imposed local measures, and nuanced differences in flow which require further qualitative exploration. This provides specific targets for understanding of both the ongoing pandemic response and planning for future health system shocks.

Overall, we demonstrate that network analysis is a novel and effective tool to describe systems behavior with an ability to identify opportunities for intervention with targeted improvements, such as strategically locating and scaling resources, streamlining transfers, and ultimately providing patient-centered approaches to drive increased value. Importantly, it allows hospital networks to be designed for properties which may be in opposition. For example, is it preferable to have a planned dissociative system which operates in a predictable way, or one which is more associative and complex, but demonstrates a greater degree of resilience in terms a given node failing? While these answers will vary between hospitals and their particular settings, we show that network science provides a powerful tool for healthcare planners seeking to manage these difficult trade-offs.
